# Budd-chiari syndrome and renal arterial neurysms due to behcet disease: a rare association

**DOI:** 10.11604/pamj.2015.21.84.3917

**Published:** 2015-06-03

**Authors:** Abdussamet Batur, Meltem Dorum, Hasan Ali Yüksekkaya, Osman Koc

**Affiliations:** 1 Department of Radiology, Yuzuncuyil University Dursun Odabas Medical Center, Van, Turkey; 2 Necmettin Erbakan University, Meram School of Medicine, Meram, Konya

**Keywords:** Behcet´s disease, Budd-Chiari syndrome, renal arterial microaneurysms, aneurysm rupture

## Abstract

Behcet's disease is a multisystemic vasculitis of unknown etiology with a chronic relapsing course. Vasculitis in Behcet's disease with predominant vascular involvement is the only vasculitis that affects both arteries and veins of any size. Involvement of the renal artery and inferior vena cava is rare among the arteries and veins, respectively. When disease affect the veins, it is in the form of thrombosis. Arterial complications include aneurysms, stenosis and occlusions. Both rupture of arterial aneurysm and occlusion of suprahepatic veins, causing Budd-Chiari syndrome, are associated with a high mortality rate. Vascular involvement is more common in male patients than in female patients. Men and patients with a younger age of onset present with a more severe prognosis. In this case report, we describe a very rare cause of intrarenal arterial aneurysm's rupture with previous Budd-Chiari syndrome due to Behcet's disease and successful angiographic embolization of actively bleeding aneurysm.

## Introduction

Behcet's disease (BD) is a multisystemic vasculitis of unknown etiology with a chronic relapsing course, characterized by genital ulcers, oral apthous ulcers, uveitis, and occasionally vasculitis [1]. Vasculitis in Behcet's syndrome can affect both arteries and veins of all sizes [2, 3]. Venous involvement exceeds the rate of arterial involvement as reported in different studies [3]. Arterial involvement is occurs in only 1 to 7% of patients, and involvement of renal arteries is very rare among them [2, 4]. When disease affect the veins, it is in the form of thrombosis. Arterial complications include aneurysms, stenosis and occlusions [2, 3, 5]. Rupture of an arterial aneurysm is the leading cause of death. We describe here a very rare cause of intrarenal arterial aneurysm's rupture with previous Budd-Chiari syndrome due to Behcet's disease and successful angiographic embolization of actively bleeding aneurysm.

## Patient and observation

**Figure 1:**
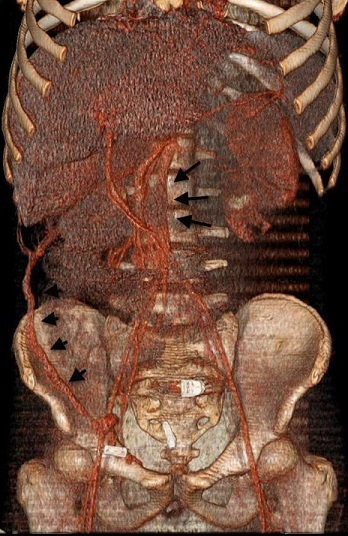
VRT images of CT angiography demonstrates vena cava inferior thrombosis (long arrows) with venous collaterals (short arrows)

Sixteen year-old male presenting with severe abdominal pain and swelling on the left quadrant of the abdomen. In the history, when he was 6 years old investigated with abdominal pain, ascites and fever. Doppler ultrasonography and BT angiography demonstrated heterogenity in liver parenchyma, hepatic vein and vena cava inferior thrombosis with venous collaterals (Figure 1). Acute phase markers were elevated. Screening revealed that the first allele of MEFV gene V726 A mutation was positive. His Factor 5 Leiden G1691A mutation, MTHFR mutation, prothrombin 20120A, lupus anticoagulan screens were negative. Protein C-S and homocysteine levels were in normal range. He was diagnosed Budd-Chiari Syndrome with Familial Mediterranian Fever. Colchicine treatment had been started. At the age of 8 years he presented with iliopsoas muscle hematom. Skin rash appeared during the hospital stay. Skin biopsy was consistent with leukocytoclastic vasculitis. Pathergy test was 6/5 positive. Eye examination was normal. They thought the diagnosis of Behcet´s disease, and continued to colchisine therapy. On his family history there was no kindship between the parents. His aunt was died from chronic renal failure (its etiology was not explained). In the physical examination; the height was 170cm (%25-50), weight was 55kg (%10-25), overall condition was poor, hypotensive, tachypneic, tachycardic. There were hepatosplenomegaly and swelling on the left quadrant of the abdomen. The other system examinations were normal.

**Figure 2:**
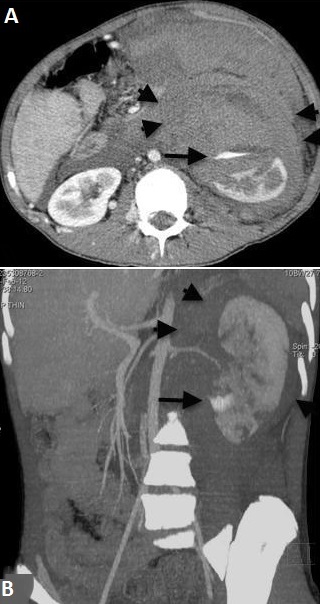
Axial (A) and 3D reconstructed coronal (B) images of the contrast-enhanced computed tomography demonstrates hematoma in the left retroperitoneal region associated with the kidney (short arrows) and signs of contrast leveling compatible with active bleeding (long arrows)

**Figure 3:**
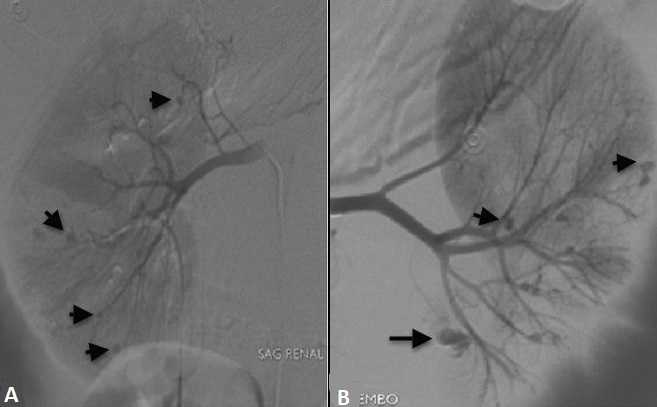
Renal angiography demonstrates multiple microaneurysms originating from the right (A) and the left (B) renal artery branches (short arrows). Left kidney localized aneurysm demonstrates irregularity consistent with rupture (long arrow)

**Figure 4:**
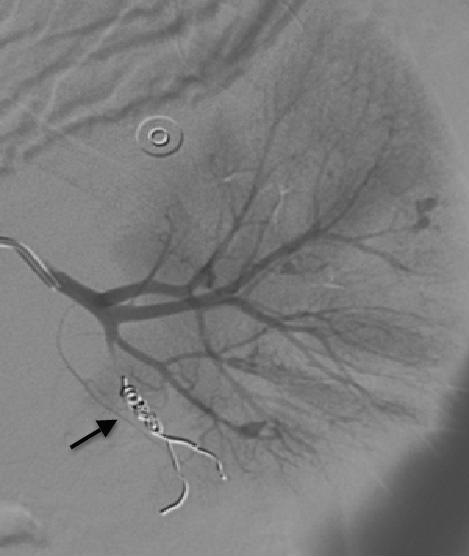
Renal angiography demonstrates no blood flow to the ruptured aneurysm after coil embolization (arrow)

The laboratory values; hemoglobine: 8.5 g/dL, platelets 228000/mm3, WBC 25.000/ul. Biochemistry and coagulation parameters were normal. Acute phase reactans were elevated (fibrinogen 533 mg/dl, erythrocyte sedimentation rate 24 mm/h, C reactive protein 29 mg/L, procalcitonine 1.064 ng/ml). In abdominal USG and tomography, in the left peritoneal region hematoma associated with kidney and signs of active bleeding was detected in the left kidney parenchyma (Figure 2). Renal angiography showed the left and right renal artery including multiple aneurysms (Figure 3). Celiac artery and superior mesenteric artery injections showed no pathology. Left inferior lobe segmental artery embolization was performed successfully (Figure 4). Which looked for the diagnosis of polyarteritis nodosa (PAN), anti-neutrophilic cytoplasmic antibody (ANCA) was positive. Steroid, cyclophosphamide and azothiopurine treatment has been started in addition to colchicine.

## Discussion

Behcet's disease with predominant vascular involvement is the only vasculitis that affects both arteries and veins of any size [3]. Patients with vasculo-Behcet are at risk of developing recurrent vascular lesions and are prone for progressive multifocal vessel related complications [2]. Aorta is the most susceptible site to aneurysm, followed by the pulmonary artery, femoral artery, subclavian artery, cervical artery and popliteal artery [6]. Involvement of the coronary and renal arteries is very rare [4]. The veins that most commonly affected are the femoral and popliteal veins [3]. Our patient presented with previous Budd-Chiari syndrome history and bilateral multiple renal artery aneurysm with one of them actively bleeding.

Vascular involvement is more common in male patients than in female patients. Men and patients with a younger age of onset present with a more severe prognosis, as it was in our case [7]. Venous and arterial involvement is characteristic. Visceral involvement is rare while large artery involvement is the most common, and inferior vena cava occlusion, causing Budd-Chiari syndrome, presents rarely among vein involvement. Both rupture of arterial aneurysm and occlusion of suprahepatic veins, causing Budd-Chiari syndrome, are associated with a high mortality rate [4, 7].

Uppon admission, our patient had a abdominal pain, swelling on the left quadrant of the abdomen and was diagnosed with non-traumatic hematoma of the kidney. There was also actively bleeding ruptured aneurysm on CT images. Zhang et al reported that there was no BD in 165 cases as a cause of non-traumatic renal bleeding [6]. To our knowledge, there have been no reports of BD both with Budd-Chiari syndrome and multiple microaneurysms in the kidney presented with renal hematoma, and superselective catheterization and embolization of actively bleeding aneurysm, with the same patient. The presence of microaneurysm in our patient could have resulted from another vasculitis, such as polyarteritis nodosa, which can primarily affect small and medium sized renal arteries. PAN could not be ruled out because of ANCA positivity.

An increased awareness of BD's vascular involvement is essential. Especially acute arterial complications should be regarded as a medical emergency. Although it is difficult to uncover the microaneurysms in the kidney on sonography or cross-sectional images, in patients with BD manifesting non-traumatic back pain, vascular involvement and bleeding of renal artery should be considered as a possible cause. Therefore, we raise the question of whether it is necessary to screen those patients with BD, using angiographic techniques for investigation of visceral microaneurysms.

Selective embolization of bleeding aneurysm has the advantage of being a minimally invazive and live-saving with the salvage of the kidney, as in our case. Vena cava inferior, hepatic vein thrombus and positive pathergy test supports Behcet Disease and in addition the other medical treatment colchicine treatment is still ongoing.

## Conclusion

Acute arterial involvement in Behcet's should be regarded as a medical emergency, and acute abdominal pain with non-traumatic hematoma of the kidney should be considered as a possible cause. Endovascular stent-grafting is a treatment of choice even for selected patients with Behçet's disease with aneurysms.
